# Histopathology of brain AVMs part II: inflammation in arteriovenous malformation of the brain

**DOI:** 10.1007/s00701-020-04328-3

**Published:** 2020-04-18

**Authors:** Roosa Wright, Patrik Järvelin, Henri Pekonen, Sara Keränen, Tuomas Rauramaa, Juhana Frösen

**Affiliations:** 1 Hemorrhagic Brain Pathology Research Group, NeuroCenter, Kuopio University Hospital, Kuopio, Finland; 2 AIV-Institute, University of Eastern Finland, Kuopio, Finland; 3 Department of Pathology, Kuopio University Hospital and University of Eastern Finland, Kuopio, Finland; 4 Hemorrhagic Brain Pathology Research Group, University of Tampere, Tampere, Finland; 5 Department of Neurosurgery, Tampere University Hospital, Teiskontie 35, PoBox 33521, Tampere, Finland

**Keywords:** Arteriovenous malformation, Rupture, Microhemorrhage, Vascular degeneration, Inflammation, Brain

## Abstract

**Background:**

Hemorrhage from an arteriovenous malformation of the brain (bAVM) has been associated with focal inflammation of the bAVM. Intrigued by the possibility of anti-inflammatory drug therapy to stabilize bAVMs and prevent hemorrhage, we investigated the association of bAVM inflammation with other histological features and clinical presentation.

**Materials and methods:**

Tissue samples from 85 surgically treated bAVMs were studied with histology and CD45 immunostainings. The histological data was compared with the clinical history of the patient. Univariate analysis and logistic regression were performed.

**Results:**

Inflammation was found in all studied bAVMs and did not associate with rupture (*p* = 0.442). While multiple types of inflammatory cells were present, macrophages were clearly the dominant inflammatory cell type, especially in samples with strong inflammation (87% of the samples). Of those bAVMs that had strong inflammation, only 56% had presented with clinically evident rupture. However, hemosiderin which is a sign of prior hemorrhage was detected in 78.4% (58/74) of samples with strong inflammation and was associated with it (*p* = 0.003). Inflammation in the nidus and parenchyma was associated with perivascular inflammation (*p* < 0.001). Multivariate analysis did not reveal any independent histological or clinical risk factor for inflammation.

**Conclusions:**

Since strong inflammation is present in both unruptured and ruptured bAVMs, it is not just a reaction to rupture. Our observations suggest that inflammation of the bAVM may indeed predispose to fragility and hemorrhage of the nidal vessels. Further studies in the role of inflammation in the untreated clinical course of bAVMs are indicated.

**Electronic supplementary material:**

The online version of this article (10.1007/s00701-020-04328-3) contains supplementary material, which is available to authorized users.

## Introduction

Arteriovenous malformations of the brain (bAVMs) are rare vascular anomalies that may rupture causing disabling or even fatal intracranial hemorrhages [2, 3, 7, 8, 29]. BAVMs arise from dysregulation of angiogenesis, which in most sporadic bAVMs can be explained by the presence of a somatic activating KRAS mutation [22] but may also be caused by other mutations [9]. Most bAVMs remain asymptomatic until their discovery, which happens as a consequence of sudden intracerebral hemorrhage (ICH) [8, 15].

It is still unclear which molecular and cellular mechanisms cause the destabilization and rupture of these lesions. Previous hemorrhage is the strongest risk factor for subsequent hemorrhage, whereas association with other factors such as sex, location, treatment, deep draining veins, and nidal volume has not been consistently replicated [3, 4, 7, 8, 14, 27, 29]. The prevalence of bAVM in the general population is approximately 1/100,000 [2] and hemorrhage is the most common clinical manifestation of bAVM which eventually occurs in approximately 52% of patients [10]. For unruptured brain AVMs, the average hemorrhage rate is estimated to be around 1–3% per year [10, 14, 17, 33]. Brain AVMs are a leading cause of fulminant hemorrhage in children and young adults [2]. Their rupture and resulting intracranial hemorrhage are associated with significant morbidity and mortality [2, 3, 7, 8, 31]. Other neurological manifestations of bAVMs include headache, seizures, pain, weakness, and problems with speech, vision, or movement.

Prior studies indicate that inflammation plays a fundamental role in the progression and rupture of bAVMs. Inflammation can cause weakening of the vessel walls, which may lead to vascular instability and can make the bAVM more prone to rupture [6, 20]. Multiple inflammatory gene promoter polymorphisms have been associated with not only the development of bAVMs but also with their rupture [18, 23]. High levels of angiogenic factors, upregulated also during inflammation [24], have also been shown to significantly contribute to bAVM destabilization and rupture [5, 13]. Previous studies have reported higher levels of inflammatory cells in ruptured bAVMs [19, 21], and abnormally high numbers of macrophages have been detected in the brain parenchyma as well as around the vascular walls in also the unruptured and untreated bAVMs [12]. These findings suggest that inflammation of the bAVM vessels may make the lesion more susceptible to hemorrhage by enhancing abnormal vascular remodeling and weakening the nidal vessels in previously stable bAVMs [1, 11, 12, 35].

In this study, we investigated in surgically treated bAVM tissue samples how inflammation associates with bAVM rupture or with clinical or histological variables.

## Materials and methods

### Clinical data

BAVM patients treated surgically between 1983 and 2018 at Kuopio University Hospital (KUH) were retrospectively identified from institutional databases using bAVM and ICD-10 diagnoses Q28.2 and Q28.3 as search terms. A total of 85 formalin-fixed paraffin-embedded (FFPE) bAVM tissue samples collected during surgery for diagnostic purposes were identified from the archives of the Department of Pathology and included in the study. The study was approved by the Ethics Committee of the Hospital District of Northern Savo.

Clinical data was collected from the patients’ medical records. Variables used in the study are presented in Table 1. Outcome after surgery was assessed using the modified Rankin Scale (mRS). Outcomes were classified as favorable (mRS score 0–3), unfavorable (mRS score 4–5), or death (mRS score 6).

**Table 1:** Association of patient demographics and clinical presentation with inflammation

Clinical variable	Inflammation (*n* = 85)		
	Grade 1	Grade 2 or 3	*p* value
Age (median and range)	25.0 years (4–67)	35.5 years (4–73)	0.231
Sex (% of females)	45.4% (5/11)	41.9% (31/74)	0.823
Smoking	0.0% (0/11)	10.8% (8/74)	0.252
Diabetes	0.0% (0/11)	2.7% (2/74)	0.581
Hypertension	0.0% (0/11)	9.5% (7/74)	0.287
Ruptured bAVM	81.8% (9/11)	55.4% (41/74)	0.106
Epilepsy (before surgery)	27.3% (3/11)	33.8% (25/74)	0.668
Embolization	36.4% (4/11)	56.8% (42/74)	0.205
Prior radiotherapy	0.0% (0/11)	9.5% (7/74)	0.287

### Histology and immunohistochemistry

For histological analysis, 4-μm sections were cut, deparaffinized, and rehydrated using standard protocols. These sections were stained with hematoxylin-eosin (HE) as well as with anti-CD45 immunostaining. For the anti-CD45 immunostaining, the sections were deparaffinized, followed by antigen-retrieval in heated citrate buffer (pH 6), and 30 min serum block in 3% normal horse serum in PBS. After the serum block, the sections were incubated with anti-CD45 mouse monoclonal antibody (clone 2B11 + PD7/26, DAKO, Glostrup, Denmark) diluted 1:100 in 1.5% normal horse serum in PBS at 4 °C overnight. Following 3 × 5 min PBS washes, the sections were incubated 30 min at RT with a biotinylated anti-mouse secondary antibody (Vectastain, Vector, Burlingame, CA, USA; 1:200 dilution). After this, sections underwent 3 × 5 min washes in PBS, a 20-min endogenous peroxidase block with 3% H2O2 in PBS, a second 3 × 5 min wash in PBS, followed by 30 min incubation with horseradish peroxidase conjugated avidin-biotin complex. Peroxidase activity signifying bound primary antibody was detected with diaminobenzidine (DAB). The sections were counterstained with hematoxylin and mounted with Depex after dehydration. Sections with primary antibody omitted were used as negative controls.

Stained tissue sections were scanned with a digital slide scanner (Nanozoomer XR, Hamamatsu, Japan), following which all the scanned sections underwent histological analysis using NDP.view2 software and up to × 80 magnification when necessary.

### Histological analysis

The specimens were scored by three independent observers (HP, PJ, RW), following which consensus score was attained and reviewed by a neuropathologist (TR). The histological variables which were scored are summarized in Table 2. Definitions for the scoring criteria are given in supplemental Table 1. The histological variables were rated on a binary scale (0 = no, 1 = yes) with the exception of inflammation, hemorrhage, and hemosiderin which were rated on a 4-point ordinal scale. The scoring scale which was used for assessing inflammation is described in supplemental Table 2.

**Table 2:** Association of histological presentation with inflammation

Histological variable	Inflammation (*n* = 85)		
	Grade 1	Grade 2 or 3	*p* value
Immature vessels	72.7% (8/11)	82.4% (61/74)	0.442
Hyalinized vessels	9.1% (1/11)	20.3% (15/74)	0.376
Perivascular inflammation	0.0% (0/11)	56.8% (42/74)	0.000
Microvascular hemorrhage	81.8% (9/11)	85.1% (63/74)	0.776
Hemosiderin	36.4% (4/11)	78.4% (58/74)	0.003
Macrophages	36.4% (4/11)	83.8% (62/74)	0.000
Neutrophils	81.8% (9/11)	81.1% (60/74)	0.953
Immature vessels	72.7% (8/11)	82.4% (61/74)	0.442
Hyalinized vessels	9.1% (1/11)	20.3% (15/74)	0.376

### Statistical analysis

Chi-square and Fisher’s exact test were used for categorical data and Mann–Whitney *U* test for continuous variables. Logistic regression with backward selection was used for multivariate analysis. Alpha level was 0.05. The statistics were calculated with SPSS 22.0 software (IBM Corp., Armonk, NY, USA).

## Results

### Nidal inflammation present also in unruptured bAVMs and does not associate with prior rupture

**Fig. 1:**
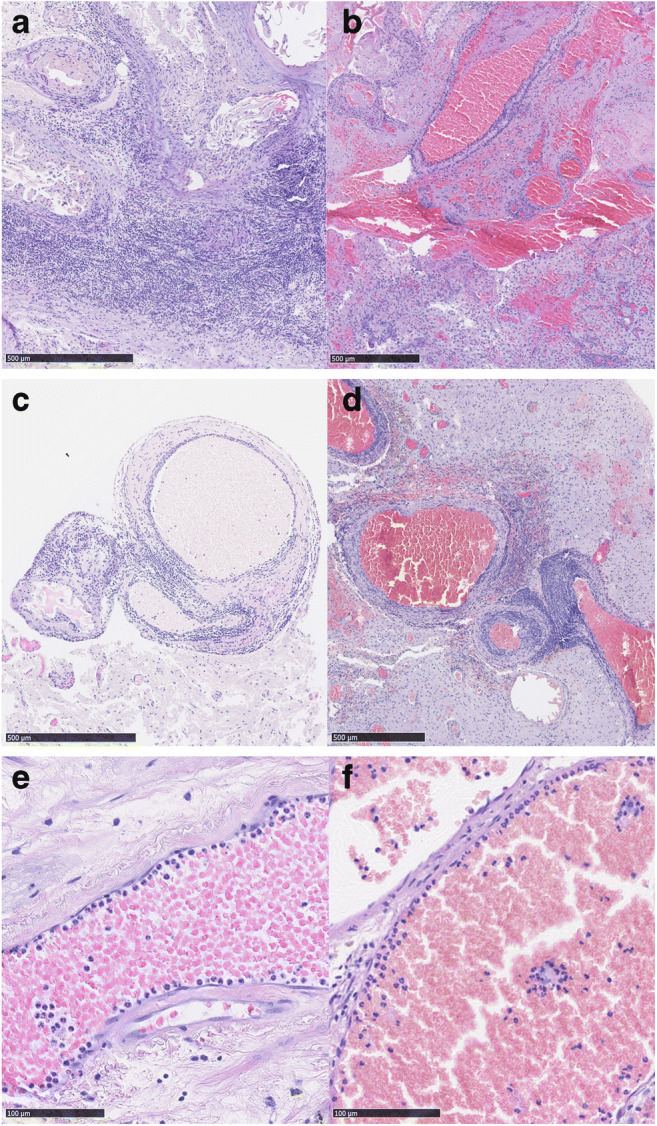
Hematoxylin and eosin staining on sections from unruptured and ruptured bAVM tissues. Strong inflammation was present in parenchyma both unruptured (**a**) and ruptured (**b**) bAVMs. In addition to inflammation of the brain parenchyme adjacent to the bAVM nidus, also, perivascular inflammation was found both in unruptured (**c**) and ruptured (**d**) bAVMs. Neutrophil adhesion and infiltration of the bAVM vessels was seen in both unruptured (**e**) and ruptured (**f**) bAVMs

Inflammation was present to some degree in all our samples (Fig. 1). Strong inflammation (grade 2 or 3) was present in 87.1% of the samples. Infiltration of inflammatory cells into brain parenchyma and bAVM vessel walls was clearly observed both in ruptured and unruptured bAVMs. Multiple types of inflammatory cells were present, including neutrophils, eosinophils, macrophages, and lymphocytes. Inflammation in bAVM tissue sample did not associate with any of the clinical variables. There was no association between prior clinically diagnosed rupture and inflammatory cell infiltration (median inflammation score 2, range 1–3 in both unruptured and ruptured bAVMs, *p* = 0.442). Of the bAVMs with strong inflammation, 55.4% (41/74, *p* = 0.106) were ruptured. A summary of patient demographics and of the clinical presentation is given in Table 1.

### Histological correlates of nidal inflammation

Parenchymal inflammation did not associate with any histological variable, such as immature or hyalinized vessels, microvascular hemorrhage, and neutrophil infiltration in the parenchyma. Inflammation in the parenchyma and perivascular inflammation were very strongly correlated (*p* < 0.001). Perivascular inflammation was found in both ruptured and unruptured bAVMs. In comparison to the specimens with slight inflammation (grade 1), we observed more macrophages in samples with strong inflammation (*p* < 0.001). Hemosiderin was detected in 78.4% (58/74) of samples with strong inflammation (*p* = 0.003). A summary of the relationship between inflammation and other histological variables is presented in Table 2.

### Multivariate analysis of the clinical and histological variables associated with inflammation

In a logistic regression model with inflammation as the dependent variable and age, sex, rupture status, hemosiderin, embolization, and macrophage infiltration as explaining variables, none of the factors was statistically significant. Perivascular inflammation was left out of the analysis due to strong association between perivascular and parenchymal inflammation. Treatment with embolization was included in the analysis due to its strong pro-inflammatory effect. Results are given in Table 3.

**Table 3:** Logistic regression analysis for association of clinical and histological variables with inflammation

	OR	95% CI	*p* value
Macrophages	3.2	0.5–19.1	0.195
Hemosiderin	3.4	0.9–13.4	0.084
Rupture status	0.4	0.1–2.3	0.292
Sex	0.6	0.1–2.6	0.478
Age	1.0	1.0–1.1	0.542
Embolization	1.3	0.3–6.4	0.739

## Discussion

In our study, inflammation was present in both ruptured and unruptured bAVM tissue samples. This implies that the inflammation present in bAVMs is not just a reaction to rupture. The observation that hemosiderin deposition, a sign of prior microhemorrhage, associated with inflammation suggests that small subclinical microhemorrhages may cause or predispose to the inflammation of the bAVM nidus parenchyme. Alternatively, inflammation in the bAVM nidus parenchyme may predispose to microhemorrhages from the nidal vessels.

### Types of inflammatory cells infiltrating the bAVM vessels and the nidus

Previously, neutrophils and macrophages have been reported in the vascular wall as well as adjacent to parenchyma, but T and B lymphocytes have been rarely observed in unruptured bAVM [6]. Neyazi et al. showed that patients with ruptured bAVM have higher levels of CEACAM1-positive immune cells with the morphology of neutrophil granulocytes compared to patients with unruptured bAVM [21]. Additionally, Li et al. reported that the matrix-degrading protease expression levels were higher in ruptured bAVM compared to the unruptured group [19]. Our data are consistent with the view that inflammation plays an important role in the brain AVM pathophysiology. CD45 staining demonstrated that infiltration of inflammatory cells was present in the brain parenchyma and the vessel walls in both ruptured and unruptured bAVMs. Moreover, in line with prior reports, we also observed polymorphonuclear cells (mostly neutrophils) in the bAVM nidus and vessel walls. Nevertheless, mononuclear cells (macrophages, T cells, B cells) were the dominant type of inflammatory cell in our samples.

### Cause of the inflammatory cell infiltration in the bAVMs—reaction to hemorrhage or something else?

Although inflammation of the bAVM did not associate with prior clinical history of rupture in our samples, response to prior hemorrhage seems nevertheless as one of the possible causes for an inflammatory response in the bAVM nidus. In a prior study by Guo Y et al., 30% of unruptured bAVMs demonstrated microscopic evidence of hemosiderin in walls of the nidal vessels [11], suggesting that a large number of bAVMs considered unruptured and stable according to their clinical presentation have in fact experienced clinically silent microhemorrhages. In our Finnish bAVM samples from KUH, parenchymal inflammation was associated with hemosiderin (a histological sign of prior hemorrhage) in the parenchyma adjacent to the nidus. These observations suggest that inflammation in the bAVM nidus is at least in part related to intralesional microhemorrhages. However, it remains to be determined whether this inflammatory cell infiltration is part of the clearance of hemoglobin breakdown products derived from the microhemorrhages [28, 32]. It also seems possible that inflammation due to other causes and subsequently increased angiogenesis and vascular permeability could be one of the underlying mechanisms predisposing to intralesional microhemorrhages. In prior studies, macrophages were present even in the hemosiderin negative specimens [12], which is in accordance with our finding that 10/23 (43.5%) of hemosiderin negative samples nevertheless had inflammatory cells (morphologically macrophages). These observations suggest that response to hemorrhage does not completely explain the macrophage infiltration in bAVMs. Furthermore, the significant presence of polymorphonuclear cells (especially neutrophils) that are not involved in the clearance of prior hemorrhage implies for another primary cause of inflammation response that may secondarily be amplified or modified by responses to hemorrhage.

### Putative clinical implications

Although our findings are inconsistent with previous studies which have reported higher levels of inflammatory cells in ruptured bAVMs [19, 21], our study along with several others confirms the presence of inflammatory cell infiltration in bAVMs, including unruptured ones [6, 12]. This in turn implies that inflammation plays a significant role in the pathobiology, evolution, and clinical course of bAVMs.

Activated inflammatory cells infiltrating tissues such as the bAVM can produce and secrete several types of molecules, such as cytokines, myeloperoxidase, MMPs, and other proteolytic enzymes that can destabilize vascular lesions [20], leading to rupture of, e.g., the bAVM nidus. Our recent report from this same series of bAVM samples shows that inflammatory cell infiltration in the bAVM vessel walls is associated with the presence of microhemorrhages in the nidal vessels [16]. Considering inflammation as a potential cause for intranidal hemorrhage, of great interest is the observation that neutrophils adhering to the luminal surfaces of bAVM nidal vessels (60%, 51/85 of samples) which strongly associated with microhemorrhage. Recruited neutrophils in bAVMs have been shown to secrete proteolytic enzymes, such as myeloperoxidase, cytokines, and matric metalloproteinases (MPPs), causing damage to the vessel walls. Particularly, the presence of MPPs has been shown to be associated with neutrophils, as myeloperoxidase (an enzyme most abundantly expressed in neutrophils) and MMP-9 have been shown to co-localize in bAVMs [20]. MPPs have been linked with vascular destabilization and altered angiogenesis [26] and plasma levels of MMP-9 have been shown to be elevated in bAVM patients [30], suggesting that MMPs have a role in the growth and rupture of bAVMs. Since this chain of events likely has an impact on bAVM rupture risk, MPP-driven vessel wall remodeling and the causes behind neutrophil recruitment in bAVM vessels merit further study.

Currently, four treatment options exist for bAVMs: microsurgical resection, embolization, radiosurgery, and conservative treatment. Microsurgery is the most definite way of eliminating the risk of rupture, but the localization and size of the lesion may render safe microsurgery impossible. Endovascular embolization, which aims to occlude feeding arteries and nidal vessels of the bAVM by delivering liquid embolic agents with a microcatheter, is associated with a risk of inadvertent occlusion of vessels not related to the bAVM. In addition, when left incomplete due to technical or anatomical challenges, incomplete embolization may in fact increase rupture risk [34]. In recent years, stereotactic radiosurgery (SRS), which targets a high dose of radiation at the malformation with the aim of inducing radiation-related necrosis and obliteration of the bAVM, has become a powerful treatment tool, especially for smaller bAVMs [25]. The use of effective doses in SRS is, however, limited by lesion size and the effect of SRS comes with a significant delay of several years during which time the patient is exposed to risk of rupture. With all the available treatment options having limitations and bAVMs being highly heterogenous lesions, there has been no clear consensus on how bAVMs should be treated. What is clear, however, is that some bAVMs cannot be treated safely and effectively with any of the current treatment options. Thus, there is a need for novel therapies, such as drug therapy, which would reduce the bAVM size or stabilize the lesion. Based on our results and other published studies on the role of inflammation in bAVMs, anti-inflammatory drug therapy seems worth investigating as a tool which could reduce the risk of bAVM progression and rupture. Rational development of such novel therapies requires in-depth understanding of the pathobiology and evolution of bAVMs. Such knowledge is also required to develop novel diagnostic tools which help better estimate the risk of rupture and hence the need for treatment in sporadic, asymptomatic bAVMs. To gain the necessary understanding of the pathobiology and evolution of bAVM, experimental models replicating the different molecular pathogenesis of bAVMs are needed. These models will eventually also confirm whether inflammation is a causal mediator predisposing to bAVM rupture, or whether in bAVMs inflammation is an epiphenomenon related to other biological processes ongoing in the bAVM or in the adjacent tissue.

## Conclusions

We showed that inflammation observed in bAVMs is not just a reaction to the prior rupture, but instead strong inflammation is found also in unruptured bAVMs. Furthermore, our results imply that inflammation may predispose to hemorrhage of the nidal vessels. The role of inflammatory cells as a source of matrix-degrading proteases and mediators of vessel remodeling in bAVMs merits further studies.

## Electronic supplementary material

ESM 1 (DOCX 13 kb)
