# Supplementary material for: Histopathology of brain AVMs part II: inflammation in arteriovenous malformation of the brain
Source: Acta Neurochir (Wien). 2020 Apr 18;162(7):1741–7. doi: 10.1007/s00701-020-04328-3 (PMC7295713; doi:10.1007/s00701-020-04328-3)
Supplement: Supplementary file 1 — (DOCX 13 kb) [file 701_2020_4328_MOESM1_ESM.docx]

Supplemental data for Wright et al. “**Inflammation in arteriovenous malformation of the brain. A putative target for drug therapy.”**

Supplemental Table 1. Definition of histological variables.

| *Histological variable* | *Definition* |
| --- | --- |
| Hemorrhage | The presence of bleeding in the brain parenchyma. |
| Inflammation | The presence of inflammation in the parenchyma. |
| Neutrophils | The presence of neutrophils in the parenchyma and neutrophil infiltration in the intima or media of the vessel. |
| Macrophages | The presence of macrophages in the parenchyma. |
| Eosinophils | The presence of eosinophils in the parenchyma. |
| Perivascular inflammation | Inflammatory cells clustered around vessels. |
| Immature vessels | Thin-walled vessels with a single layer of endothelial cells, large enough to have multiple red blood cells next to each other (too large to be capillary). |
| Hyalinized vessels | The presence of hyalinized vessels. |
| Hemosiderin | The presence of hemosiderin in the parenchyma. |
| Microvascular hemorrhage | The presence of bleeding around a small vessel. |
| Calcification | Deposition of calcium in the vessel wall. |
| Embolization material | The presence of embolization material in the vessel wall. |
| Necrosis | The presence of necrosis in the parenchyma. |

Supplemental Table 2. The grading system used to describe inflammation in the bAVMs.

| grade 0 | No detected inflammatory cells in the brain parenchyma. |
| --- | --- |
| grade 1 | Inflammatory cells detected in the parenchyma with a magnification higher than 1.25x. |
| grade 2 | <30% of the area covered by inflammatory cells, can be seen with a magnification of 1.25x. |
| grade 3 | >30% of the area covered by inflammatory cells, can be seen with a magnification of 1.25x. |
